# Patients treated for infection following ACL reconstruction with graft removal have poorer outcomes than those treated with graft retention: A systematic review

**DOI:** 10.1002/jeo2.70147

**Published:** 2025-03-06

**Authors:** Daniel C. Lewis, Natalya E. McNamara, Erin M. Tabish, Joseph T. Featherall, Hillary W. Rawson, Gregoire Micicoi, Daniel J. Song, Justin J. Ernat

**Affiliations:** ^1^ Department of Orthopedic Surgery University of Utah Health Salt Lake City Utah USA; ^2^ School of Medicine University of Utah Salt Lake City Utah USA; ^3^ Unité de Recherche Clinique Côte d'Azur (UR2CA) Université Côte d'Azur Nice France; ^4^ Sports Medicine Division Evans Army Community Hospital Fort Carson Colorado USA

**Keywords:** ACL reconstruction, graft removal, graft retention, infection, septic arthritis, systematic review

## Abstract

**Purpose:**

The purpose of this study was to evaluate patient outcomes following anterior cruciate ligament reconstruction (ACLR) complicated by septic arthritis treated with graft retention versus graft removal protocols. Secondarily, this study aimed to evaluate surgical, demographic and microbial surgical indications for graft retention versus graft removal. We hypothesised that patients who underwent graft removal would have worse outcomes and that patients with septic arthritis caused by more virulent organisms, such as methicillin‐resistant *Staphylococcus aureus* or *Pseudomonas aeruginosa*, would be more likely to undergo graft removal.

**Methods:**

A systematic review and meta‐analysis of literature in the PubMed and Ovid databases regarding the treatment of septic arthritis following ACLR reporting graft retention versus graft removal was conducted. The included studies were published in English, in peer‐reviewed journals, with an average minimum follow‐up of 1 year, and reported on arthroscopic ACLR, surgical management of infection, graft retention versus graft removal during treatment and outcome measures. Patient demographic, surgical and outcome data were analysed.

**Results:**

Twenty‐four studies reporting on 307 patients were included for analysis. Patients who underwent allograft ACLR (*p* = 0.02) and patients with septic arthritis caused by *P. aeruginosa* (*p* = 0.03) were more likely to undergo graft removal. Patients treated with graft removal were treated with more irrigation and debridement procedures (2.7 ± 0.8 vs. 2. ± 1.5, *p* < 0.01). Patients treated with graft removal had increased laxity on KT‐1000 measurement (3.30 ± 134 vs. 1.55 ± 1.23, *p* < 0.01), and lower 2000 International Knee Documentation Committee Subjective Knee Evaluation scores (66.57 ± 17.08 vs. 80.18 ± 15.21, *p* = 0.02).

**Conclusions:**

Septic arthritis following ACLR is a devastating complication. Both graft retention and graft removal protocols have been reported and are viable options. Patients treated with graft removal had poorer outcome measures. Septic arthritis caused by *P. aeruginosa* and allograft ACLR were more likely to be treated with graft removal.

**Clinical Relevance:**

Septic arthritis following ACLR remains an uncommon, but difficult problem. There is minimal literature guiding graft retention versus graft removal treatment protocols.

**Level of Evidence:**

Level IV systematic review of lower‐level studies.

**Abbreviations**
ACL: anterior cruciate ligament
ACLR: anterior cruciate ligament reconstruction
BTB: bone‐patellar tendon‐bone autograft
CI: 95% confidence interval
HS: hamstring autograft
IKDC: 2000 International Knee Documentation Committee Subjective Knee Evaluation
I&D: irrigation and debridement
MINORS: methodological index for non‐randomised studies
OA: osteoarthritis
OR: odds ratio
QT: quadriceps tendon autograft
ROM: range of motion

## INTRODUCTION

Septic arthritis is a rare but life‐altering complication of anterior cruciate ligament reconstruction (ACLR). There are many well‐described sequelae that can result from septic arthritis following ACLR, including additional surgeries, increased pain, decreased return to sport, increased complications with secondary surgeries like arthroplasty, secondary graft failure, diminished patient‐reported outcomes and early development of osteoarthritis (OA) [22, 27, 42, 46]. Further, septic arthritis following ACLR is associated with a substantial financial cost to the healthcare system, with a median cost of $19,356 and costs as high as $64,463 [34].

ACLR is an increasingly common procedure. From 2002 to 2014, the incidence of ACLR increased by 22%, from 61.4 to 74.6 per 100,000 patient years [13]. The rates of septic arthritis following ACLR vary in the literature but are commonly reported at around 0.6% [48, 49].

The potential severe and long‐term effects of septic arthritis following ACLR and the increasing incidence of ACLR performed have propagated scientific investigation on the risk factors and management of this complication [42]. Several studies have examined graft type as a risk factor for postoperative infection and have reported that ACLR with hamstring autograft (HS) has the highest infection rates [2, 3, 23, 24] There have been proposed management strategies for septic arthritis following ACLR [45, 46, 49]. However, overall, there remains significant variability in clinical practice and, consequently, a lack of cohesive evidence regarding the management of septic arthritis following ACLR. The purpose of this study was to evaluate patient outcomes following ACLR complicated by septic arthritis treated with graft retention versus graft removal protocols. Secondarily, this study aimed to evaluate surgical, demographic and microbial risk factors for graft retention versus graft removal. We hypothesised that patients with septic arthritis caused by more virulent organisms, such as methicillin‐resistant *Staphylococcus aureus* or *Pseudomonas aeruginosa*, would be more likely to undergo graft removal and would have worse outcomes. Additionally, we hypothesised that patients who underwent graft removal would have worse outcomes.

## METHODS

This investigation was registered with Preferred Reporting Items for Systematic Reviews and Meta‐analyses (PROSPERO, registration number CRD42022367537). Study eligibility criteria included available in the English language, publication in a peer‐reviewed journal, average minimum follow‐up of 1 year, arthroscopic treatment of ACLR, surgical management of infection with at least one irrigation and debridement (I&D), studies reporting graft retention versus graft removal during treatment, and studies reporting outcomes measures. Studies were excluded if they were non‐English, an abstract/presentation, a case report of a single patient, published in a nonpeer‐reviewed journal, reported an average follow‐up of less than 1 year, review articles, or did not report any outcome measures. Studies that reported on distinct infection outbreaks or clusters due to known contamination or failures in sterilisation were excluded. Further, studies were excluded that did not report analysable outcome data for patients who underwent treatment with graft removal and/or graft retention. All studies and data that were not attributable to patients undergoing graft retention or graft removal were excluded.

**Figure 1:**
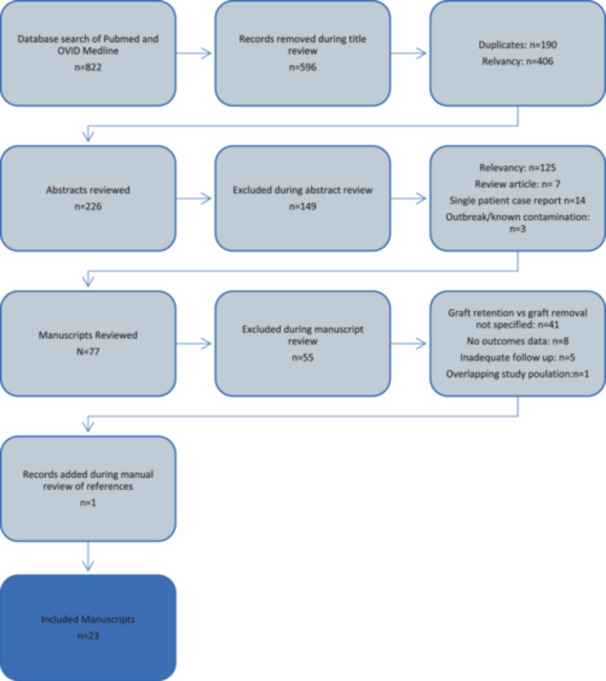
PRISMA flowchart of included and excluded reviewed works.

The PubMed and Ovid databases were queried on 10/3/2022 for a period up to 10/2/2022, this search was updated on 3/7/24, with a new additional query over the period 10/3/2022 to 3/7/2024. We queried the databases using the search criteria (‘anterior cruciate ligament reconstruction’ OR ‘ACL reconstruction’) AND (‘infection’ OR ‘septic arthritis’). In addition, manual searches were performed of cited references from any eligible study based on relevance. Broad search terminology was intentionally selected to ensure a wide catchment of studies, and an initial title review eliminated a large number of irrelevant studies. A PRISMA flow chart details the search strategy used in Figure 1.

Eligible studies were reviewed for reporting of surgical treatment protocol and summary outcome measures. Data from eligible studies were extracted manually, including demographics, infection and surgical information and outcome measurements. Extracted demographic variables included age and sex. In cases where data were reported on an individual patient basis, these were collected and analysed; if individual data were not available collective descriptive statistics were collected. Infection information collected included culture results and time to diagnosis. Surgical data collected included graft type, graft retention versus graft removal at the time of I&D and the number of procedures performed to treat septic arthritis. Objective outcome measurements included were range of motion (ROM) and KT‐1000 knee arthrometer measurement versus contralateral (MEDmetric Corp.). Patient‐reported outcomes were also collected, including the International Knee Documentation Committee (IKDC), Lysholm and Tegner scores. Forest plots were constructed for studies where data were available reporting comparable discrete outcomes measures, given the variability in study design and methodology random effects models were utilised.

The methodological index for non‐randomised studies (MINORS) was used to appraise manuscripts and assess the risk of bias in and across the studies [43]. Two authors reviewed all manuscripts and any disagreement was resolved by meaningful discussion with a third author. Criteria were graded as described in previous studies [30, 31, 38]. High quality met >75%, moderate quality 50%–74%, low quality 25%–49% and very low quality <25% of the MINORS criteria. The strength of evidence of the pooled studies also was guided by descriptions used in previous studies and rated as ‘strong’, ‘moderate’, ‘limited’, ‘very limited’ or ‘conflicting’ [10, 38, 51, 52].

All statistical analysis was conducted in Microsoft Excel 16.82 (2024, Microsoft Corporation) and R version 4.3.2 (2023, The R Foundation for Statistical Computing). Data reported on an individual patient basis as well as data reported collectively from study groups were utilised. In cases where mean and standard deviation values necessary for meta‐analysis were not reported in the original publications, accepted methods for estimating mean and standard deviation from median, range or interquartile range as applicable based on originally reported data were used [14, 26, 47]. Chi‐square test was utilised to analyse categorical values where all groups had greater than five patient sample size, the Fisher Exact test was used to analyse categorical values where at least one group had five or fewer patients, and Student's *t* test was utilised for continuous variables. Effect size was reported as standardised mean difference for continuous variables and odds ratio (OR) for categorical variables. The *I* ^2^ statistic was used to assess heterogeneity between studies that reported on comparable outcome measures. The threshold for significance was set at a *p* value of <0.05.

## RESULTS

Twenty‐three (23) studies met inclusion criteria with a total of 269 patients [1, 3, 4, 5, 8, 11, 12, 15, 16, 18, 25, 27, 28, 29, 32, 36, 37, 39, 41, 44, 50, 53]. The ACL graft was retained throughout treatment in 233 (86.6%) patients. The ACL graft was removed in 36 (13.4%) patients (Table 1). Patients who did and did not undergo revision ACLR, as well as those where revision ACLR was not reported, were included in the graft removal group. Revision ACLR was reported in 4 patients, while no revision ACLR was specified in 10 patients, individual information regarding revision ACLR was not reported for the remaining 22 patients. The mean age was 29.5 years (range, 13–56) at the time of index surgery. Patients were mostly male, 81.7%, while 18.3% were female.

**Table 1:** Patients treated with graft retention and graft removal in the included studies.

Study	Level of evidence	Graft retention patients	Graft removal patients	Total patients
Pogorzelski et al. [36]	IV	21	12	33
Lo Presti et al. [37]	IV	16	0	16
Monaco et al. [29]	IV	12	0	12
Windhamre et al. [5]	III	27	0	27
Abdel‐Aziz et al. [1]	III	24	0	24
Barker et al. [3]	IV	13	5	18
Binnet et al. [4]	IV	6	0	6
Burks et al. [6]	IV	0	4	4
Calvo et al. [8]	IV	5	2	7
Fong et al. [11]	IV	7	0	7
Gobbi et al. [12]	IV	7	0	7
Indelli et al. [15]	IV	4	2	6
Judd et al. [16]	IV	10	1	11
Kim et al. [18]	IV	6	1	7
McAllister et al. [25]	IV	4	0	4
Meglic et al.	II	18	0	18
Mishra et al. [28]	IV	17	0	17
Sajovic et al. [39]	IV	3	0	3
Van Tongel et al. [44]	IV	11	0	11
Williams et al. [50]	IV	3	4	7
Zalavras et al. [53]	IV	0	5	5
Musso et al. [32]	IV	9	0	9
Schollin‐Borg et al. [41]	III	10	0	10
Total		233	36	269

MINORS analysis was conducted (Table 2). Most studies were of moderate quality (14 out of 23) [4, 6, 8, 16, 18, 25, 29, 32, 36, 37, 39, 44, 50, 53], while six studies were high quality [1, 5, 15, 27, 28, 41] and three were low quality [3, 11, 12]. Few studies included prospectively collected data [1, 27, 28]. Only two studies included prospective calculation of study size, and all other studies made no mention of calculation of study size [1, 5]. One study reported adequate methods for ensuring unbiased end point data analysis [27]. We recognise, however, that although 6 of the studies scored as ‘high quality’ and 14 studies scored as ‘moderate quality’ using MINORS criteria, 19 out of 23 studies reviewed were Level IV, retrospective case studies [3, 4, 6, 8, 11, 12, 15, 16, 18, 25, 28, 29, 32, 36, 37, 39, 44, 50, 53]. Thus, defining these findings as ‘moderate’ based on previously used definitions may be overstating the findings.

**Table 2:** MINORS analysis of included studies.

Study	1	2	3	4	5	6	7	8	9	10	11	12	Total	% Criterion met	Quality
Pogorzelski et al. [36]	2	2	1	2	1	2	1	0					11	69%	Moderate
Lo Presti et al. [37]	2	2	1	2	1	2	1	0					11	69%	Moderate
Monaco et al. [29]	2	2	1	2	1	1	2	0					11	69%	Moderate
Boström Windhamre et al. [5]	2	2	1	2	1	2	1	2	2	2	2	2	21	88%	High
Abdel‐Aziz et al. [1]	2	2	2	2	1	2	2	2	2	2	2	2	23	96%	High
Barker et al. [3]	2	2	1	2	1	0	0	0					8	50%	Low
Binnet and Başarir [4]	2	2	1	2	1	2	2	0					12	75%	Moderate
Burks et al. [6]	2	2	1	2	1	2	1	0					11	69%	Moderate
Calvo et al. [8]	2	2	1	2	1	2	0	0					10	63%	Moderate
Fong and Tan [11]	2	2	1	1	1	1	0	0					8	50%	Low
Gobbi et al. [12]	2	2	1	0	0	0	2	0					7	44%	Low
Indelli et al. [15]	2	2	1	2	1	2	2	0					12	75%	High
Judd et al. [16]	2	2	1	2	1	2	1	0					11	69%	Moderate
Kim et al. [18]	2	2	1	1	1	2	2	0					11	69%	Moderate
McAllister et al. [25]	1	2	1	2	1	2	2	0					11	69%	Moderate
Meglic et al. [27]	2	2	2	2	2	2	2	0	2	2	2	2	22	92%	High
Mishra et al. [28]	2	2	2	2	1	2	2	0					13	81%	High
Sajovic et al. [39]	1	2	1	2	1	1	2	0					10	63%	Moderate
Van Tongel et al. [44]	2	2	1	2	1	2	1	0					11	69%	Moderate
Williams et al. [50]	1	2	1	2	1	1	2	0					10	63%	Moderate
Zalavras et al. [53]	2	2	1	2	1	1	2	0					11	69%	Moderate
Musso and McCormack [32]	2	2	1	2	1	1	2	0					11	69%	Moderate
Schollin‐Borg et al. [41]	2	2	1	2	1	2	2	0	2	2	2	2	20	83%	High

*Note*: Items 1–12 represent: (1) a clearly stated aim; (2) inclusion of consecutive patients; (3) prospective collection of data; (4) end points appropriate to the aim of the study; (5) unbiased assessment of the study end point; (6) follow‐up period appropriate to the aim of the study; (7) loss to follow up less than 5%; (8) prospective calculation of the study size; (9) an adequate control group; (10) contemporary groups; (11) baseline equivalence of groups; and (12) adequate statistical analysis. The item scored 0 indicates not mentioned, 1 indicates reported but inadequate and 2 indicates reported and adequate. Maximum score of 16 for a self‐controlled study; maximum score of 24 for a controlled study.

Age was not found to be significantly different between the graft removal and graft retention groups (*p* = 0.98). Additionally, there was no statistically significant difference in sex between the graft retention and graft removal groups (*p* = 0.10) (Table 3).

**Table 3:** Patient demographics in the graft retention and graft removal groups.

	Graft retention	Graft removal	*p*‐value
Total patients, *n* (% of total)	258 (84.0%)	49 (16.0%)	
Female sex, *n* (%)	29 (16.3%)	10 (28.6%)	0.10
Age, mean ± standard deviation (years)	29.5 ± 8.5	29.5 ± 12.3	0.98
Hamstring graft	141	25	0.02
Patellar tendon (BTB) graft	34	4	
Quadriceps tendon graft	1	0	
Allograft	4	5	
Time to diagnosis, mean ± standard deviation (days)	19.3 ± 34.1	25.9 ± 19.2	0.10
Number of procedures, mean ± standard deviation	2.1 ± 1.5	2.7 ± 0.8	<0.01

HS was the most utilised graft at the index procedure (77.6%), followed by bone‐patellar tendon‐bone autograft (BTB) (17.8%), allograft (4.2%) and quadriceps tendon autograft (QT) (0.5%). Of the allografts, five were antibiotic and gamma radiation‐treated Achilles tendon allografts; the type of graft and treatment protocol was not reported for the other four allografts. There was a statistically significant difference in graft type between graft removal and graft retention treatment groups (*p* = 0.02, OR = 2.02) (Table 3). Patients who underwent allograft ACLR were significantly more likely to be treated with graft removal than those with autograft ACLR (*p* < 0.01).

The diagnosis of septic arthritis following ACLR was determined clinically from patient symptoms and laboratory evaluation. On average, diagnosis was made approximately 3 weeks after index surgery, but time to diagnosis varied widely (20.2 ± 34.1 days; range, 1–95 days). There was no difference in time to diagnosis between graft retention and graft removal groups (*p* = 0.10) (Table 3).

Culture results were reported in 266 cases (86.6%). Cultures were obtained from a postoperative joint aspiration or intraoperatively at the time of I&D. A wide variety of organisms were isolated (Table 4). Coagulase‐negative *S. aureus* was the most isolated organism, followed by methicillin‐sensitive *S. aureus* (48.5% and 24.8%, respectively). Eighteen cases (6.7%) were polymicrobial, and no organisms were isolated on culture in 11.7% of cases where culture data were available. There were no statistically significant differences overall in treatment with graft retention or graft removal based on culture results (*p* = 0.07). However, when culture data were analysed individually, patients infected with *P. aeruginos*a were more likely to be treated with graft removal (60%) than graft retention (14.4%) versus other organisms (*p* = 0.03). There was no difference in graft removal rates in polymicrobial or culture‐negative infections (*p* = 1.00 and *p* = 0.16, respectively). There was no significant difference in the number of procedures based on the pathogen, except for *Escherichia coli*; however, this was based on only two cases (Table 5).

**Table 4:** Causative organisms for septic arthritis in the graft retention and graft removal groups.

	Graft retention	Graft removal	*p*‐value
Total culture‐positive infections	199	36	
Coagulase‐negative *Staphylococcus* species	114	15	0.10
Methicillin‐sensitive *Staphylococcus aureus*	53	13	0.31
*Cutibacterium acnes*	8	1	1.0
*Enterococcus* species	5	3	0.11
Methicillin‐resistant *Staphylococcus aureus*	5	2	0.29
*Peptostreptococcus* species	5	1	1.00
*Streptococcus* species	5	0	1.00
*Pseudomonas aeruginosa*	2	3	0.03
*Enterobacter* species	3	1	0.49
*Corynebacterium* species	3	1	0.49
*Escherichia coli*	2	1	0.39
*Klebsiella pneumoniae*	2	0	1.00
*Bacillus cereus*	1	0	1.00
*Stenotrophomonas maltophilia*	1	0	1.00
*Finegoldia magna*	0	1	0.15
Polymicrobial infections	13	5	0.16
Culture negative	28	3	0.59

**Table 5:** Number of irrigation and debridement procedures in the treatment of septic arthritis by causative organism.

	*N*	Mean number of procedures	*p*‐value
Total culture‐positive infections	154	1.85 ± 1.00	
Coagulase‐negative *Staphylococcus* species	51	1.72 ± 0.92	0.41
Methicillin‐sensitive *Staphylococcus aureus*	51	2.10 ± 1.22	0.19
*Cutibacterium acnes*	6	1.67 ± 1.03	0.47
*Enterococcus* species	6	1.83 ± 0.98	0.97
Methacillin‐resistant *Staphylococcus aureus*	6	1.83 ± 0.75	0.96
*Peptostreptococcus* species	6	2.00 ± 1.26	0.79
*Pseudomonas aeruginosa*	5	2.20 ± 1.10	0.52
*Enterobacter* species	4	1.80 ± 1.00	0.85
*Streptococcus* species	2	1.50 ± 0.71	0.61
*Escherichia coli*	2	3.00 ± 0.00	<0.01
*Klebsiella pneumoniae*	1	4	N/A
*Corynebacterium* species	1	1	N/A
Polymicrobial infections	10	1.90 ± 1.10	0.89
Culture negative	24	1.45 ± 0.51	<0.01

**Figure 2:**
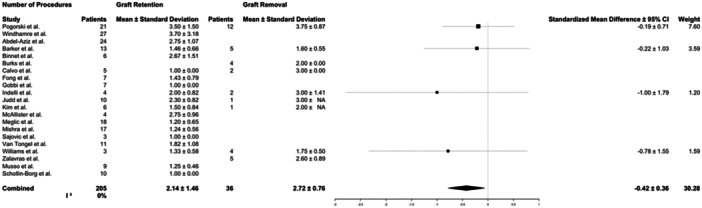
Forest plots for the number of patients and mean and standard deviations of the number of procedures for patients reported in the studies who underwent graft retention or graft removal. 95% CI, 95% confidence interval.

Similarly, there was heterogeneity in treatment protocols following the diagnosis of septic arthritis. All patients in the included studies were treated surgically with at least one I&D and antibiotics. There were no specific or consistent criteria reported for graft retention versus graft removal. In the included studies, the decision for graft removal relied on surgeon experience without specific criteria or standardised protocols utilised. Indications for graft removal included nonviable graft, significant gross infection of the graft, and failure of treatment with graft retention; however, specific criteria for these indications were not reported. Fourteen studies (171 patients) only included treatment with graft retention, while in 2 studies (9 patients) all patients were treated with graft removal. Patients underwent an average of 2.2 ± 1.6 procedures (range, 1–6), and patients treated with graft removal underwent more procedures than those with graft retention (2.7 ± 0.8 vs. 2.1 ± 1.6, respectively, *p* < 0.01) (Table 3 and Figure 2). There was little variability between studies reporting the number of procedures performed (*I* ^2^ = 0%). Revision ACLR after successful treatment of septic arthritis in patients treated with graft removal was not included in the total procedure count because it was inconsistently reported.

**Figure 3:**
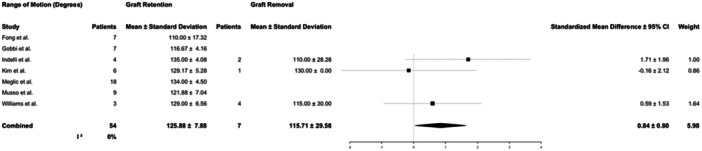
Forest plots for the number of patients and mean and standard deviation range of motion for patients reported in the studies who underwent graft retention or graft removal. 95% CI, 95% confidence interval.

**Figure 4:**
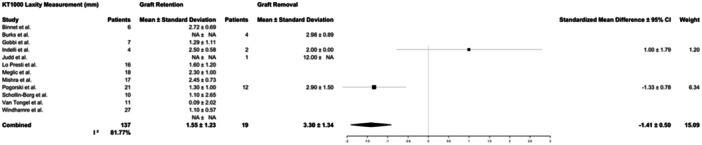
Forest plots for the number of patients and mean and standard deviation laxity measurements for patients reported in the studies who underwent graft retention or graft removal. The KT1000 testing device was used to determine laxity compared to the contralateral knee measured in mm. 95% CI, 95% confidence interval.

Final follow‐up across all studies averaged 45 months and varied widely (range, 4–196 months). Recorded objective outcome measures included knee ROM at final follow‐up and KT‐1000 testing compared to the contralateral knee. ROM was reported by 7 studies accounting for 61 patients and KT‐1000 measurements by 12 studies in 156 patients. Patients in both graft retention and graft removal groups had mildly decreased ROM compared to the contralateral side at follow‐up, with no difference in ipsilateral range of motion between graft removal and graft retention groups (126 ± 12° vs. 115 ± 25°, respectively, *p* = 0.33). KT‐1000 measures differed between groups. Patients treated with graft removal had significantly more laxity via KT‐1000 testing (3.30 ± 1.34 mm) compared to those treated with graft retention (1.6 ± 1.2 mm, *p* < 0.01) (Table 6; Figures 3 and 4). The variability between studies reporting ROM outcomes was low (*I* ^2^ = 0%), while variability for KT‐1000 measures was high (*I* ^2^ = 81.77%).

**Table 6:** Patient‐reported and objective outcomes for patients in the graft retention and graft removal groups.

	Graft retention	Graft removal	*p*‐value
Range of motion, mean ± standard deviation (degrees)	126 ± 8°	116 ± 30°	0.40
KT‐1000 difference to contralateral, mean ± standard deviation (mm)	1.6 ± 1.2	3.3 ± 1.3	<0.01
IKDC score, mean ± standard deviation	80.2 ± 15.2	66.6 ± 17.1	0.02
Lysholm score, mean ± standard deviation	81.2 ± 16.7	70.3 ± 24.4	0.07
Tegner score, mean ± standard deviation	5.6 ± 1.8	6	N/A

Abbreviation: IKDC, International Knee Documentation Committee.

**Figure 5:**
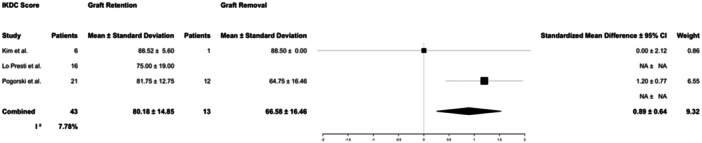
Forest plots for number of patients and mean and standard deviation International Knee Documentation Committee (IKDC) score for patients reported in the studies who underwent graft retention or graft removal. The IKDC score is a patient assessment that ranges knee scores from 0 to 100 [21]. 95% CI, 95% confidence interval.

**Figure 6:**
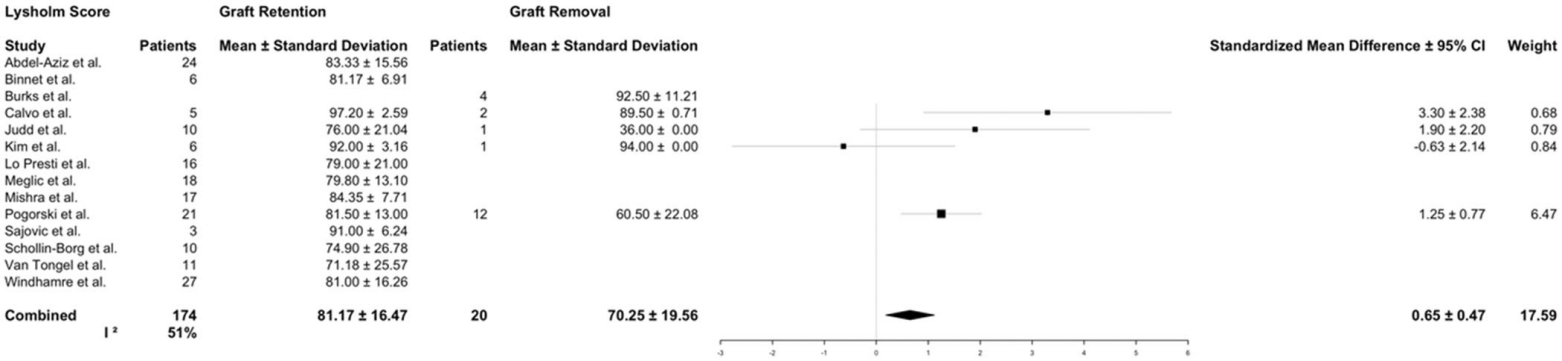
Forest plots for number of patients and mean and standard deviation Lysholm score for patients reported in the studies who underwent graft retention or graft removal. The Lysholm score is a 100‐point scoring system used to help physicians evaluate knee‐related symptoms post‐injury or surgery [21]. 95% CI, 95% confidence interval.

Patient‐reported outcome measures collected included IKDC (3 studies), Lysholm (14 studies) and Tegner (8 studies) scores. Patients who were treated with graft retention reported better outcomes than those who underwent graft removal. Patients in the graft retention group had better IKDC scores (80.2 ± 15.2 vs. 66.6 ± 17.1, *p* = 0.02) than those in the graft removal group. This difference (13.6) does meet the threshold for minimal clinically important difference that has been previously reported in cases of ACLR (9.5) [33]. Lysholm scores (81.2 ± 16.6 vs. 70.4 ± 24.4, *p* = 0.07) did not display statistically significant differences. Seven studies reported Tegner scores for patients in the graft retention group (5.6 ± 1.8); however, a Tegner score was only reported for one patient in the graft removal group (6), and therefore, comparative analysis was not performed for this patient‐reported outcome measure (Table 6; Figures 5 and 6). Variability between study reporting of IKDC score was low (*I* ^2^ = 7.78%), and variability for studies reporting Lysholm score was moderate (*I* ^2^ = 51.0%).

Limited data were available to compare patients treated with graft removal who underwent revision ACLR to those who did not undergo revision. Based on the limited data available there was no significant difference between patients who underwent revision ACLR and those who did not in IKDC scores (73.2 vs. 64.4, *p* = 0.18) or Lysholm scores (81.1 vs. 69.0, *p* = 0.81) (Table 7). Even fewer studies reported KT‐1000 laxity measurements in this population in 4 patients. A single study reported 2.3 ± 0.6 mm of increased laxity in 4 patients who underwent revision ACLR, while 4.0 ± 3.3 mm of laxity was reported in 10 patients who did not undergo revision, this difference was not statistically significant (*p* = 0.33). Additionally, there was no significant difference in KT‐1000 laxity measurement between patients who underwent graft retention and those who underwent graft removal with revision ACLR (*p* = 0.25).

**Table 7:** Patient‐reported outcomes for patients who did and did not undergo revision ACLR after septic arthritis treatment with graft removal.

	Revision ACLR	No revision ACLR	*p*‐value
Total patients with data available, *N* (% of total)	13 (41.9%)	18 (58.1%)	
IKDC score, mean ± standard deviation (*N*)	73.2 ± 16.9 (11)	64.4 ± 14.3 (15)	0.18
Lysholm score, mean ± standard deviation (*N*)	81.1 ± 15.9 (12)	69.0 ± 19.8 (17)	0.82
KT‐1000 difference to contralateral, mean ± standard deviation (mm) (*N*)	2.3 ± 0.6 (4)	4.0 ± 3.3 (10)	0.33

Abbreviations: ACLR, anterior cruciate ligament reconstruction; IKDC, International Knee Documentation Committee.

## DISCUSSION

Overall, literature reporting on septic arthritis following ACLR was heterogeneous, without large high‐quality studies or prospective trials. This systematic review showed that in terms of both objectively measured examination and patient‐reported outcomes, patients treated with graft removal have worse results compared to graft retention procedures. Further research is needed to determine whether this is due to the inferiority of graft removal treatment protocols, or worse infections requiring graft removal.

Septic arthritis following ACLR is an uncommon complication; however, it can lead to significant patient morbidity [2, 7, 22, 40, 42]. There is no consensus regarding treatment protocol for septic arthritis following ACLR, and treatment varies widely. It is generally accepted that operative I&D along with culture‐guided antibiotics is standard of care. Both arthroscopic and open I&D are commonly accepted and performed. There is not a clear consensus on removal versus retention of the ACL graft in these patients, although, in this review, most patients were treated with graft retention. Despite this, there are no clear indications for when a graft should be removed, and these decisions are based on surgeon judgement and determination of a nonviable graft. Some authors advocate for graft retention in all cases, while others always perform graft removal [6].

There are multiple systematic reviews evaluating risk factors and outcomes after septic arthritis following ACLR; however, few studies have compared the role of graft retention versus graft removal in treatment for these infections. Kursumovic et al. reported an 85% graft retention rate in infection following ACLR with HS graft but were unable to analyse outcome measures as they were not consistently reported [19]. Makhni et al. and Schmitz et al. performed reviews of patient outcomes after septic arthritis following ACLR compared to patients who did not have postoperative infections, but they did not compare graft retention and graft removal [22, 40]. Kusnezov et al. performed an expected value decision analysis, which heavily favoured initial graft removal [20]. However, this conclusion was based on volunteer ‘hypothetical patient's’ outcome preferences and outcome probabilities, and the results were most heavily influenced by patient preference to avoid late reoperation.

Patients who underwent graft removal reported significantly poorer patient‐reported outcome measures based on IKDC scores. In terms of objective postoperative assessments, there were no differences in ROM between the two groups, although, in both groups, there was a diminished range of motion compared to the unaffected knee. The KT‐1000 laxity measurements were increased after graft removal compared to graft retention; it is worth noting, however, that both patients who underwent revision ACLR after clearance of septic arthritis and those who did not were included in this analysis. There was no significant difference in KT‐1000 laxity measurements between patients who underwent revision ACLR and those who did not undergo revision ACLR after graft removal, but the numbers available were very low. These objective and patient‐reported outcomes conflict with the expected value decision analysis reported by Kusnezov favouring early graft removal, and ultimately drive a compelling argument for graft retention [20].

In other areas of orthopaedics, infections caused by *P. aeruginosa* are considered difficult to treat [9, 17, 35]. In this study, patients with *P. aeruginosa* septic arthritis were more likely to undergo graft removal. However, this study does not offer insights into the cause behind the increased rate of graft removal; this may be from surgeon bias in more aggressively treating *P. aeruginosa* or from increased graft colonisation and damage from a more virulent infection. The increased rate of graft removal is consistent with the unfavourable nature of *P. aeruginosa* in general, and culture‐proven *P. aeruginosa* septic arthritis may be an indication for more aggressive early graft removal.

Additionally, septic arthritis that occurred following allograft ACLR was more likely to undergo graft removal than ACLR with autograft. This area has been extensively studied and previously published literature has not shown higher rates of septic arthritis in allograft ACLR [2]. One might speculate that devitalised tissue, like an allograft, would carry with it a higher risk of septic arthritis early on; however, there is no data to support this. In actuality, HS autografts have historically demonstrated the highest rates of post‐ACLR septic arthritis [3, 24]. In this systematic review, those patients who received allograft ACLRs were more likely to undergo graft removal. This same reasoning could be surgeon‐biased—surgeons are warier of colonisation of devitalised tissue, or a contaminated allograft as a source of infection may be a subconsciously easier culprit to accept than a surgical error or break in sterile technique. It could also be that a greater attempt is made at saving autografts given the morbidity the patient experience during harvest. Additionally, there are additional patient factors, including age and comorbidities, that could contribute to infection severity, immune response or surgeon decision‐making, which may be confounders. Further research is certainly required.

Prospective trials would be required to further elucidate the superiority of graft retention or graft removal protocols, and specific indications for graft removal protocols. However, given the low rate of septic arthritis following ACLR organising an adequately powered trial would be extremely difficult and expensive, which is one of the reasons why this review, despite significant limitations, is valuable. Additionally, and perhaps more practically, large survey studies of current practices and expert surgeon decision‐making could shed light on why allograft ACLR are more likely to undergo graft removal and could be used to develop a decision‐making algorithm for the treatment of septic arthritis following ACLR, both of which remain open questions after this review.

### Limitations

This study has several limitations. First, the data were extremely heterogeneous both in reporting methods as well as in the surgical and medical treatment protocols of patients. Data were collected from two large online databases of peer‐reviewed literature, but it is possible that other relevant studies were not captured in this review because they were not in English or not available in the reviewed databases. Because of the heterogeneity and poor quality of the data, several compromises had to be made in patient selection and data analysis. An average minimum follow‐up of 1 year for each study population was selected as a criterion to eliminate studies that focused only on the immediate post‐operative period; however, to avoid the exclusion of large numbers of patients with adequate follow‐up, some individual patients with less than 1 year of follow‐up were included. Additionally, there were low numbers of patients in the allograft and quadriceps tendon groups, which could be a source of error. Many studies did not report values as means and standard deviations, and in these cases, estimates based on statistical calculations had to be used. However, published/accepted techniques for these estimations were utilised, which allowed for an analysis of a significantly larger cohort than what has been published about post‐ACLR septic arthritis in the past [14, 26, 47]. The low numbers and heterogeneity of the studies limit the ability of this study to conclude whether the statistically significant findings are clinically significant for patients. There is no established or widely accepted protocol for the treatment of septic arthritis following ACLR. Therefore, the patients included in this study were treated in different ways, largely based on the clinical judgement of the treating surgeon. This introduces the risk of bias based on other clinical factors besides just graft removal versus graft retention. However, this heterogeneity also highlights the importance of more high‐quality literature in this field, and hence our attempt to synthesise such with a focus on the clinical factors pertinent to the treatment of septic arthritis following ACLR. It could be viewed as a limitation that patients who did and who did not undergo revision ACLR were included in the outcomes group for graft removal, but these patients chose to undergo/forgo another surgery and ultimately their reported outcomes represent the patient experience for those who undergo graft removal.

## CONCLUSIONS

Septic arthritis following ACLR is a devastating complication. Both graft retention and graft removal protocols have been reported and are viable options. Patients treated with graft removal had poorer outcome measures. Septic arthritis caused by *P. aeruginosa* and allograft ACLR were more likely to be treated with graft removal.

## AUTHOR CONTRIBUTIONS

**Daniel Lewis**: Statistical analysis and manuscript writing/revision. **Natalya McNamara**: Conception of design and data acquisition. **Erin Tabish**: Data acquisition. **Joseph Featherall**: Statistical analysis and interpretation of the work. **Hillary Rawson**: Final approval of version and compliance with review board standards. **Gregoire Micicoi**: Drafting/revising critically for important intellectual content and final approval of version. **Daniel Song**: Drafting/revising critically for important intellectual content and final approval of version. **Justin Ernat**: Conception; interpretation; manuscript writing/revision and final approval of version to be published.

## CONFLICT OF INTEREST STATEMENT

Justin J. Ernat reports a relationship with Johnson & Johnson Depuy Mitek Sports Medicine, including consulting or advisory roles. He also reports a relationship with the Arthroscopy Association of North America and the American Orthopaedic Society for Sports Medicine, both of which include board membership. Dr. Ernat is an editorial board member for the Arthroscopy Journal and has received funding from the L.S. Peery Foundation for other research unrelated to the current topic. The other authors declare no conflicts of interest.

## ETHICS STATEMENT

Given that this is a systematic review of previously published literature, the study was deemed exempt by the Institutional Review Board.

## Data Availability

The data that support the findings of this study are openly available in the PROSPERO database at https://www.crd.york.ac.uk/prospero/display_record.php?RecordID=367537, reference number CRD42022367537.
